# Diarrheagenic *Escherichia coli* pathotypes frequency in Khuzestan province of Iran

**Published:** 2016-12

**Authors:** Atieh Darbandi, Parviz Owlia, Saeid Bouzari, Horieh Saderi

**Affiliations:** 1 Department of Microbiology, Faculty of Medicine, Shahed University, Tehran, Iran; 2 Molecular Microbiology Research Center, Shahed University, Tehran, Iran; 3 Molecular Biology Department, Pasteur Institute of Iran, Tehran, Iran

**Keywords:** *Escherichia coli*, Pathotype, Diarrhea

## Abstract

**Background and Objectives:**

Diarrheagenic *Escherichia coli* (DEC) is an emerging agent among pathogens that causes diarrhea. Studies showed that diarrheagenic *E. coli* such as enterohaemorrhagic *E. coli* (EHEC), enteroaggregative *E. coli* (EAEC), enteropathogenic *E. coli* (EPEC), enteroinvasive *E. coli* (EIEC), enterotoxigenic *E. coli* (ETEC), diffusely adhering *E. coli* (DAEC) and shiga toxin producing *E. coli* (STEC) strains are among the most frequent causative agents in acute diarrhea. The aim of this study was to determine the frequency of DEC pathotypes in Khuzestan province.

**Materials and Methods:**

Stool samples were collected from patients with diarrhea in Khuzestan province of Iran. *E. coli* strains were isolated using conventional culture and standard biochemical tests. The polymerase chain reaction (PCR) technique was used to detect presence of virulence genes, i.e; *eae*, *stx*1 and *stx*2 for EHEC, *bfp* and *eae* for EPEC, *LT* and *ST* for ETEC, *AA* for EAEC, *invE* for EIEC, *stx*1 and *stx*2 for STEC.

**Results:**

Altogether, 200 stool samples were obtained from patients, of which 158 (79%) were positive for *E. coli*. DEC was identified in 127 (63%) of stool samples, which frequency of each pathotypes were as follows: atypical EPEC 49 (39%), typical EPEC 1 (0.7%), STEC 50 (39.3%), ETEC 21 (16.3%), EAEC 5 (4.0%) and EIEC 1 (0.7%). Most frequent etiological agents of diarrhea in Khuzestan province of Iran were STEC and EPEC.

**Conclusion:**

Our findings showed DEC had been agent of diarrhea in Khuzestan. This finding provides evidence that effort should be made to estimate the burden of infection by the etiological agent for better medical approach and should raise notification about antibiotic resistance among bacterial infection.

## INTRODUCTION

Diarrhea is defined as the passage of three or more loose or liquid stools per day, according to the WHO definition, which is a major public health problem worldwide (1). The mortality rates estimated to be around 1.5–2.5 million, especially in Africa, Asia and Latin America. *Escherichia coli* is recognized as a significant cause of epidemic and endemic diarrhea in all over the world (1). At least seven categories of diarrheagenic *E. coli* are recognized on the basis of their specific virulence properties, serotypes, and different epidemiological and clinical features (2), including enteroaggregative *E. coli* (EAEC), enterohaemorrhagic *E. coli* (EHEC), enteroinvasive *E. coli* (EIEC), enteropathogenic *E. coli* (EPEC), enterotoxigenic *E. coli* (ETEC), shiga toxin producing *E. coli* (STEC), and diffusely adhering *E. coli* (DAEC). EPEC is divided into two groups, typical EPEC (tEPEC) and atypical EPEC (aEPEC). Typical EPEC strains also carry the large EPEC adherence factor (EAF) plasmid and it is absent in aEPEC (2). ETEC is the leading cause of adult traveler′s diarrhea visiting endemic areas; this pathotype produces the plasmid-encoded heat-labile enterotoxin (LT) and heat-stable enterotoxin (ST) or both (1). Among the diarrheagenic *E. coli* categories, EAEC appears to have been increasingly recognized as an emerging pathogen responsible for acute and persistent diarrhea in human (3). The majority of EAEC isolates harboring a 60–65 MDa virulence plasmid, referred to as DNA probe PCVD432 (3). EIEC has biochemical, physiological and genetic properties similar to *Shigella* species and also invade eukaryotic cells (2). The genes related to invasion are located on a 140 virulence plasmid (PInV), which encodes a type III secretion system (2). EHEC isolation is often associated with O157:H7 serotype that produces a shiga-like toxin responsible for systematic clinical symptoms such as hemorrhagic colitis and hemolytic uremic syndrome in humans (4). The main features of STEC are the bacteriophage-encoded *stx*1 and/or *stx*2 genes that determine the production of shiga toxin (*stx*) (4). This pathotype present the most important recently emerged group of food and water borne pathogens (5). DAEC is defined by a characteristic diffuse pattern of adherence to HEP-2 cell (2).

The aim of the present study was to investigate the presence and frequency of diarrheagenic *E. coli* (DEC) in adults and children with diarrhea by PCR assay in Khuzestan province.

## MATERIALS AND METHODS

### Study population

Fecal samples from 200 patients with diarrhea from all age groups in Khuzestan province (Ahwaz, Andimeshk and Shoshtar) were collected for a period of one year from March 2012 to February 2013.

### Bacteriological procedures

All specimens were cultured on MacConkey agar (Merck, Germany) and were incubated for overnight at 37°C. *E. coli* isolates were selected and confirmed by biochemical tests, including conventional lactose fermentation (using TSI medium and IMVIC test) (6). Also, different culture methods for screening fecal specimens for *E. coli* O157:H7 were used such as MacConkey agar (MAC) containing sorbitol instead of lactose (SMAC) (Merck, Germany) and CHROM agar (CHROMagar, France) (4).

### DNA extraction

All *E. coli* isolates strains were grown on Luria-Bertani broth (Sigma, USA) overnight at 37°C and DNA was extracted using the alkaline-lysis method (7).

### Detection of virulence genes by PCR

The PCR for identification of eight virulence genes of distinct DEC groups was used. The specific oligonucleotide primers used in this study are shown in Table 1. The optimized protocol was carried out with a 20 μL mixture containing 10 μL master mix (2×) (Fermatas, Lithuania) that contain Taq DNA polymerase (0.05 U/μL), reaction buffer, 4mM MgCl_2_ and 0.4 mM of each dNTP and 7 μL deionized water, 2 μL of two primers and 1 μL DNA extract. The condition for PCR were 94°C for 5 min (initial denaturation of DNA within the sample) followed by 30 cycles of 94°C for 1 min (denaturation), 50°C to 63°C (see Table 1) for 1 min (primer annealing), and 72°C for 1 min (DNA synthesis) and final DNA synthesis at 72°C for 10 min, which performed with a thermal cycler (Eppendorf, USA).

**Table 1.** Primers used for detection of genes indicative of particularpathotype

**Gene**	**Oligonucleotide Sequence (5′–3′)**	**Conc (μM)**	**Fragment Size (bp)**	**Annealing temp (° C)**	**Reference**
*Elt*	GAACAGGAGGTTTCTGCGTTAGGTG CTTTCAATGGCTTTTTTTTGGGAGTC	0.1	655	63	7
*Estla*	CCTCTTTTAGTCAGACARCTGAATCASTTG CAGGCAGGATTACAACAAAGTTCACAG	0.4	157	63	7
*aggR*	CTGGCGAAAGACTGTATCAT CAATGTATAGAAATCCGCTGTT	0.2	457	63	8
*Eae*	AGGCTTCGTCACAGTTG CCATCGTCACCAGAGGA	0.4	570	59	9
*bfpB*	GACACCTCATTGCTGAAGTCG CCAGAACACCTCCGTTATGC	0.1	910	63	7
*Stx*1	CAATGCCACGCTTCCCAGAATTG GATGTTACGGTTTGTTACTGTGACAGC	0.2	244	63	9
*Stx*2	GTTTTGACCATCTTCGTCTGATTATTGAG AGCGTAAGGCTTCTGCTGTGAC	0.4	324	63	9
*invE*	CGATAGATGGCGAGAAATTATATCCCG CGATCAAGAATCCCTAACAGAAGAATCAC	0.2	766	63	7

### Positive and negative controls

All reference strains were from personal collection in the molecular biology department of Pasteur Institute, Tehran, Iran. *E. coli* K12 was used as negative control for all genes in PCR assay. Positive controls for each gene were as follows: *E. coli* O157:H7 for *eae, stx*1 and *stx*2; E2348/64 for *bfp*; E17.2 for *aggR*; H10407 for *elt* and *estla*; *Shigella*12022 for *invE.*

## RESULTS

A total of 158 out of 200 isolate (79%) were *E. coli*. Distribution of various pathotypes is shown in Table 2. Overall 127 (63%) out of 158 *E. coli* was detected as different pathotypes. We found that aEPEC, tEPEC, STEC non-O157:H7, ETEC, EAEC and EIEC were 49 (39%), 1 (0.7%), 50 (39.3%), 21 (16.3%), 5 (4%) and 1 (0.7%), respectively. The most frequent category of diarrheagenic *E. coli* detected were STEC 50 (39.3%) and aEPEC49 (39%).

**Table 2.** Distribution of isolated DEC pathotypes according to age and gender

**DEC**	**Gene**	**No. positive patients (%)**	**Age groups (years)**			**Gender**	

			**<5**	**5–14**	**>14**	**Male**	**Female**
aEPEC	*eae*	49(39)	14	9	26	23	26
tEPEC	*bfp*	1(0.7)	-	1	-	-	1
	*stx*1	5(4)	1	1	3	4	1
	*stx*2	11(8.6)	2	4	5	4	7
	*stx*1*, stx*2	2(1.5)	1	1	-	1	1
STEC	*stx*1*, eae*	17(13.4)	5	3	9	13	4
	*stx*2*, eae*	4(3.2)	1	-	3	2	2
	*stx*1*, stx*2*, eae*	11(8.6)	2	1	8	4	7
	*lt*	3(2.3)	1	1	1	3	-
ETEC	*st*	17(13.3)	4	6	7	7	10
	*lt, st*	1(0.7)	1	-	-	-	1
EAEC	*aggR*	5(4)	1	-	4	3	2
EIEC	*InvE*	1(0.7)	-	-	1	1	-
Total		127	33	27	67	65	62

In our study, 11 samples were positive for three genes (*eae, stx*1, *stx*2), hence checked for EHEC pathotype, but after cultured on SMAC and CHROM agar, they were categorized in non-O157 STEC. In addition, in STEC pathotype, the *stx*1 with *eae* were detected as the most frequent gene (13.4%). The *stx*1 and *stx*2 were detected in only 1.5% of the STEC pathotype isolates. *Elt* gene was detected in 3 (2.3%) isolates, while the *Estla* gene was detected in 17 (13.3%) and only one in isolate both *Elt* and *Estla* genes were detected.

**Fig. 1.**
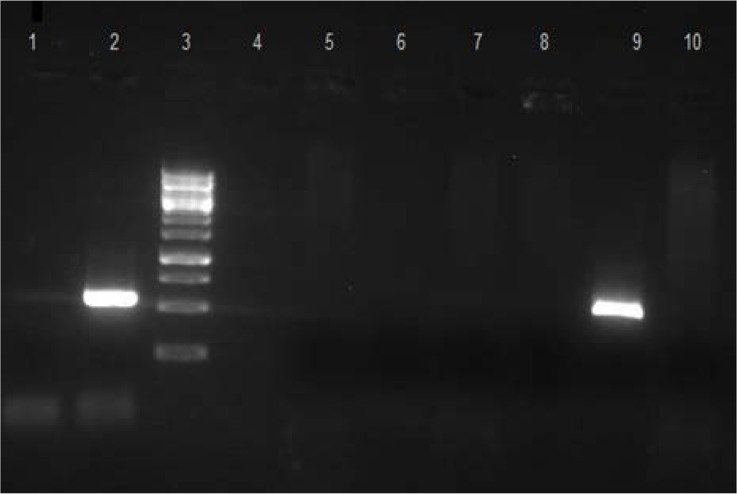
Agarose gel electrophoresis of PCR product, showing presence of *eae* gene. Lane1, K12 (Negative control); lane 2, O157:H7 (Positive control) (570bp); lane 3, DNA molecular size markers (1 kb ladder); lanes 4–8 and 10, patient samples (without *eae* gene); lane 9, patient sample (with *eae*).

The distribution of different pathotypes among various age groups revealed that EPEC and STEC were highly frequent in age group higher than 14 years (53% and 56%, respectively). However ETEC was frequent in all age group (Table 2). As far as the sex of patients was concerned, there was no difference between different age groups and pathotypes (Table 2). Altogether, 42% of pathotypes occurred in winter, followed by spring, summer and autumn, respectively (Table 3).

**Table 3.** Seasonal distribution of different isolated DEC pathotypes

**Season**	**No. positive patients (%)**					

	**EPEC**	**STEC**	**ETEC**	**EAEC**	**EIEC**	**Total**
Winter	20(40)	25(50)	5(24)	3(60)	-	53(42)
Spring	14 28)	10(20)	4(20)	1(20)	-	29(23)
Summer	6(12)	10(20)	6(28)	1(20)	-	23(18)
Autumn	10(20)	5(10)	6(28)	-	1(100)	22(17)

## DISCUSSION

The present study demonstrated frequency of different pathotypes of *E. coli* with diarrhea in Khuzestan province, southwest of Iran. In the present study, the rate of isolation of pathotypes was 63%. In another study from Iran, diarrheagenic *E. coli* (ETEC, EPEC, EAEC and STEC) were detected in 38.8% (10). In South India and Sweden, isolation rate of different pathotypes in acute diarrhea were reported to be 52% and 56%, respectively (11, 12). While the rate was 4.8% in Korea, lower than other parts of the world (13). In our study, 5 different pathotypes (EPEC, STEC, ETEC, EAEC and EIEC) were identified in various frequencies.

STEC includes different serotypes, among them O157:H7 is the most important serotype (14). The present result did not reveal any instances of the O157:H7 serotype in our setting. However, there is a report by Salmanzadeh-Ahrabi et al. (14). It should be mentioned that for isolation of O157:H7 accurate and specific test should be performed in the reference laboratories (8). In the present study, a high frequency (39%) of STEC was detected in 200 screened patients. Another study in Iran, Salmanzadeh-Ahrabi et al. reported that STEC is an important cause of acute diarrhea (14). This result showed that non-O157 STEC were the major cause of human infections in this area of Iran and also non-O157 STEC were also isolated in other countries like Germany, Italy and Denmark with higher frequency than O157:H7 strains (15). Non-O157 STEC may also play an important role in disease compared to STEC O157:H7 as shown in Argentina, Australia and Chile (15) while, in Canada, United States, Japan, England and Scotland, the frequency of non-O157 is very low (16). Among identified dietary risk factors, foods of bovine origin, particularly undercooked ground beef, have been a frequently implicated source. Non-dietary risk factors, including person-to-person transmission in day-care settings or swimming in contaminated water have also been documented (17). For rapid and sensitive detection of non-O157 STEC strains, PCR has proven to be best for detection, especially for the detection of *stx* genes (18). In our study, most of the isolates belonged to STEC, and >70 per cent of STEC were positive for *stx*1 gene. Aslani et al. showed a large proportion of isolates (96.5%) possessed the gene for *stx*1 (18).

Among the isolates of diarrheagenic *E. coli*, aEPEC were high frequent (39%), as also was found in study conducted in Chile (38.3%) (19). However, a low frequency of EPEC was observed in Kenya (7.4%) (20). Isolation of only one typical EPEC strain confirms a recent trend that has been observed in the study of Gomes et al. (21). In recent reports, however, from different countries as diverse as Poland, South Africa, United Kingdom and Australia, atypical EPEC strains have outnumbered typical strains as a cause of gastroenteritis (22, 23). These findings were reflected in the present study; 49 (39%) of *eae*-bearing strains identified in patients with gastroenteritis were atypical EPEC. In general, atypical EPEC were originally incriminated as intestinal pathogens by virtue of their epidemiologic association with cases of diarrhea (24).

ETEC had been relatively frequently isolated (16.3%), similar to study in Korea, which ETEC was detected in 17% of cases (25). These results are similar to several studies reported from elsewhere (Evans et al., 1977) (26), but different from Bangladesh (Sack et al. 1977) (27). The importance of this pathogen in diarrheal disease in Iran has not been studied extensively, perhaps because the complex biological assays needed to detect enterotoxins. Variation in the frequency of toxin types among different geographic areas is reported. Shahrokhi et al. showed that ST-only with a 60.3% rate was the most frequent toxin type followed by LT-only (31.3%) and LT/ST (8.4%)(28). Dominance of the ST-expressing ETEC has been documented in Egypt, Bangladesh, and Iran (28, 29).

The prevalence of EAEC isolates (4.0%) was similar to study contented in Libya (4.1%), but different from that reported in Tunisia (11.3%) (30, 31).Variation in the frequency may be due to the significant geographic area and the frequencies of virulence factor (8). In this study, we used the gene *aggR* for our study, this gene in Bouzari et al. study was reported to be as 66% (32).

We diagnosed only one EIEC by PCR using *InvE* gene, similar to Levine et al. study (19) and believe that EIEC strains are relatively uncommon in Iran. Also, in Brazil (33), PCR technique was found to be very sensitive and rapid but some other tests are needed to confirm the EIEC.

In conclusion, the frequency of different pathotypes was variable in this study and some pathotypes were more frequent than the others. The results of this study supported the fact that the occurrence of *E. coli* O157:H7 was very low but non-O157 STEC was the major cause of human infections in Iran similar to study of Bonyadian (18, 34). Our findings point to the role of DEC in causing diarrhea. For the rapid detection of clinical strains, PCR has proven to be of great diagnostic value (14, 24, 32, 35). However, other confirmatory and serological tests are needed for accurate identification. Complementary studies to identify pathotypes in other provinces can help to adopt necessary measures to tackle possible outbreaks in our country.
